# Is age a critical indicator for determining the optimal abstinence duration in patients undergoing sperm acquisition?

**DOI:** 10.3389/frph.2026.1921505

**Published:** 2026-08-18

**Authors:** Yifu Wang, Shenghui Zhu, Houbin Zheng, Hai Lin, Zhiyong Zhu, Huang Liu, Yu Zhou

**Affiliations:** 1 Department of Andrology, NHC Key Laboratory of Male Reproduction and Genetics, Guangdong Provincial Reproductive Science Institute (Guangdong Provincial Fertility Hospital), Guangzhou, Guangdong, China; 2 Clinical Laboratory, NHC Key Laboratory of Male Reproduction and Genetics, Guangdong Provincial Reproductive Science Institute (Guangdong Provincial Fertility Hospital), Guangzhou, Guangdong, China

**Keywords:** abstinence duration, age, sperm acquisition, sperm cryopreservation, sperm quality

## Abstract

**Introduction:**

Sperm cryopreservation is increasingly recognized as a key strategy for fertility preservation, particularly for male infertility patients who may benefit from assisted reproductive technology (ART). However, the optimal abstinence period for maximizing semen quality prior to cryopreservation remains unclear, and whether this optimum varies with patient age has not been systematically investigated.

**Methods:**

We retrospectively analyzed semen parameters from male patients who underwent sperm cryopreservation for fertility preservation at Guangdong Provincial Reproductive Hospital. Patients were stratified by age (<40 years vs. ≥40 years). Semen samples were collected after different abstinence durations (0–2, 3–5, 6–7 and ≥7 days). Sperm quality indicators—including volume, concentration, motility, and progressive motility—were compared across abstinence groups within each age stratum.

**Results:**

Results showed that among patients aged over 40 years, sperm quality was optimal after 0–2 days of abstinence despite reductions in semen volume and concentration. By contrast, in younger patients (under 40 years), the highest semen quality was observed following 3–5 days of abstinence. Additionally, prolonged abstinence periods were linked to unstable semen quality in the older patient group, making them less ideal candidates for sperm cryopreservation.

**Discussion:**

This study is the first to demonstrate that the relationship between abstinence duration and sperm quality is strongly modified by patient age. Our findings indicate that older patients achieve better and more consistent outcomes with shorter abstinence. The findings offer new perspectives for guiding sperm cryopreservation and determining the optimal timing for sperm retrieval in patients of different age groups to improve sperm quality and increase success rates in assisted reproductive outcomes.

## Introduction

Sperm cryopreservation serves as a critical approach for men seeking to preserve fertility. Recent advances in cancer therapeutics have considerably improved long-term survival rates among male patients with cancer. However, these treatments can compromise reproductive function. As outlined in the American Society of Clinical Oncology guidelines, cryopreserving sperm prior to initiating cancer therapy represents an effective strategy for protecting male patients' future fertility (1). In addition to adult males, this method is currently being investigated in pre-adolescent boys as a strategy for preserving fertility potential into adulthood (2). Numerous studies have confirmed the efficacy of sperm cryopreservation. For example, a systematic review and meta-analysis demonstrated that this method is feasible and effective for preserving fertility in patients with cancer (3). While the utilization rate of sperm cryopreservation may be underestimated, its role in assisted reproductive technology (ART) remains substantial for patients with cancer because it provides a viable option for preserving their potential to pursue parenthood in the future (3). Sperm cryopreservation serves as a vital strategy for fertility preservation, extending beyond oncology to other clinical scenarios. For instance, transgender individuals pursuing gender-affirming interventions may choose to cryopreserve sperm before initiating hormone therapy, thereby preserving future reproductive possibilities (4). In the treatment of male infertility, sperm cryopreservation is used for not only preserving fertility but also reducing potential genetic risks to offspring. Research indicates that as the father's age increases, fertility declines and the genetic risks to the offspring increase. These issues can be effectively avoided by cryopreserving sperm at a younger age (5).

Prior to sperm cryopreservation, semen quality evaluation is essential because it directly affects the efficacy of the freezing process and the subsequent success of assisted reproductive procedures. A thorough evaluation of semen quality before freezing and after thawing prior to semen cryopreservation is crucial. Notably, men with infertility frequently show a remarkable reduction in sperm survival post-cryopreservation. Semen analysis outcomes are also strongly affected by the length of sexual abstinence. An abstinence period that is too short can result in insufficient semen volume, whereas an excessively prolonged period may reduce sperm motility. Therefore, careful attention should be given to the abstinence interval in semen assessment.

The duration of abstinence has a considerable effect on various sperm quality parameters. Short abstinence periods, such as 1 day or less, are associated with reduced sperm DNA fragmentation, indicating that brief abstinence may help minimize DNA damage and improve sperm quality (6, 7). In addition, short abstinence periods may increase the total antioxidant capacity of sperm, thereby lowering oxidative stress-related damage (8). By contrast, longer abstinence periods are generally correlated with higher sperm concentration and total sperm count. However, this increase in quantity does not necessarily reflect improved quality because extended abstinence can result in decreased sperm motility and impaired movement ability (9, 10). Some studies indicate that prolonged abstinence increases the risk of sperm DNA fragmentation, which may adversely affect fertility outcomes (11).

Furthermore, the duration of abstinence may exert differential effects across patient subgroups. For example, in men with normal semen parameters, prolonged abstinence may enhance sperm concentration and total sperm count. Meanwhile, among patients with abnormal semen parameters, a shorter abstinence period could be more advantageous for improving sperm quality, particularly motility and morphology (9, 12). Notably, the second semen sample collected following a short abstinence period has been found to exhibit substantially improved total sperm motility and progressive motility, which, in turn, correlates with enhanced clinical pregnancy rates and embryo quality (13). Consequently, individualizing the abstinence period on the basis of the patient's specific condition may serve as an effective strategy to optimize sperm quality and improve fertility outcomes. In summary, the influence of abstinence duration on sperm quality is multifaceted and subject to inter-individual variation. Although shorter abstinence may reduce DNA fragmentation and enhance sperm motility, longer abstinence tends to increase sperm quantity. Therefore, rationally determining the abstinence interval in accordance with the patient's clinical profile and reproductive objectives is crucial when performing semen analyses or fertility interventions, thereby maximizing semen quality and the likelihood of successful conception (14, 15).

In semen analysis, patient age is recognized as a key factor influencing sperm quality. With advancing age, men exhibit notable alterations in semen parameters and overall sperm quality. A negative correlation has been observed between age and conventional semen parameters, including semen volume, sperm concentration, motility, and the percentage of progressively motile sperm (16). Computer-aided sperm analysis (CASA) further indicates that the kinematic parameters of sperm are adversely affected by age (16). Additional investigations utilizing motile sperm organelle morphological examination to assess age-related effects have demonstrated a marked decline in the proportion of morphologically normal sperm and a considerable increase in sperm with large nuclear vacuoles as age increases (17). These morphological changes are associated with sperm DNA damage, suggesting that advanced paternal age may contribute to fertilization abnormalities or pregnancy loss (17). Moreover, age substantially compromises sperm DNA integrity. Older men show a pronounced increase in the sperm DNA fragmentation index (DFI), which inversely correlates with sperm concentration, total sperm count, motility, and normal morphology (18). A study conducted in China revealed that the effect of age is more substantial on sperm DNA integrity than on conventional semen parameters, with a notable increase in DNA fragmentation particularly evident in men over 35 years of age (19).

In summary, the relationship between abstinence duration and age in relation to sperm quality is multifaceted, encompassing aspects such as semen parameters, sperm morphology, and DNA integrity. To the authors' knowledge, no systematic or comprehensive analysis has been conducted regarding the optimal selection of abstinence duration protocols for patients of different age groups undergoing sperm cryopreservation. In this study, a comprehensive evaluation of semen quality in patients experiencing pre-ejaculatory fluid emission was performed, examining the influence of patient age and abstinence duration on sperm parameters. The results showed that in patients over 40 years of age, sperm quality was optimal following an abstinence period of 0–2 days despite reduced semen volume and sperm concentration. By contrast, among patients under 40 years, the highest semen quality was observed after 3–5 days of abstinence. Furthermore, extended abstinence periods were associated with unstable semen quality in patients over 40 years, rendering them less suitable for sperm cryopreservation. These findings offer guidance for tailoring abstinence periods in accordance with patient age in the context of sperm cryopreservation, thereby improving the chances of obtaining sperm in optimal condition and supporting the success of subsequent assisted reproductive treatments.

## Method

### Data collection

After men diagnosed with “fertility issues” upon visiting Guangdong Provincial Reproductive Hospital (Guangdong Provincial Institute of Reproductive Sciences) from January 2023 to December 2024 were screened, a total of 945 patients who underwent sperm cryopreservation were finally enrolled. Comprehensive physical examination and medical history collection were conducted for each patient before semen preservation. Decline in sperm quality due to other disease factors (e.g., a history of mumps, cryptorchidism, and endocrine system diseases such as adrenal gland tumors and pituitary tumors) and obvious factors (e.g., history of testicular trauma, recent history of genitourinary tract infection, and history of sauna exposure) that considerably affect male fertility were excluded. This study was approved by the Ethics Committee of Guangdong Provincial Reproductive Science Institute (Guangdong Provincial Fertility Hospital, Approval No. IIT20260504).

### Sperm analysis

In accordance with the World Health Organization's Laboratory Manual for the Examination and Processing of Human Semen (5th Edition, 2010), semen samples were obtained through masturbation in a sterile environment following a period of abstinence ranging from 2 days to 7 days. In light of practical clinical factors such as the need for time-sensitive disease treatments (e.g., initiation of cancer radiotherapy or chemotherapy) or individualized clinical requirements among some patients, this study additionally generated and incorporated a subgroup of semen samples obtained following 0–2 days of sexual abstinence. The collected samples were liquefied in a 37 °C water bath and subsequently analyzed using the CASA system (Microptic SL, Barcelona, Spain). Sperm volume was determined using the weighing method, and CASA was employed to evaluate additional parameters. Each sample underwent immediate analysis in a counting chamber, where at least 200 sperm were assessed across five microscopic fields. The recorded parameters included total sperm count, concentration, motility, and progressive motility. Sperm exhibiting a path velocity exceeding 5 mm/s were classified as motile, whereas those with a velocity greater than 25 mm/s and a linearity above 80% were designated as “progressively motile.” These parameters were aligned with the standards outlined in the World Health Organization's Laboratory Manual for the Examination and Processing of Human Semen (Fifth Edition), which specifies sperm volume at 1.5 mL, sperm count at 39 million, sperm concentration at 15 million/mL, total motility at 40%, and progressive motility at 32%. Sperm morphology was analyzed using Papanicolaou staining.

### Statistical power calculation

To assess whether the current sample size has sufficient statistical power to detect clinically meaningful effect differences, this study used G*Power 3.1 software for *a priori* sample size estimation. Based on previous literature reports, the mean sperm DFI in men with normal fertility is 12.1%, with a standard deviation of 9.8% (20). Based on the measured sperm DFI data from cryopreserved patient samples within our center, the mean DFI of this population was estimated to be 17.0%. With a two-tailed significance level (*α*) set at 0.05 and statistical power (1 − *β*) at 0.80, *a priori* sample size calculation was performed using the “One-sample t-test: Difference from constant” module in G*Power 3.1 software, yielding a minimum required sample size of 34 cases. Considering an anticipated 10% dropout rate, the target enrollment sample size was finalized at 38 cases. A total of 945 patients were actually enrolled in this study, which far exceeds the estimated minimum sample size and thus sufficiently ensures statistical power. For patients undergoing ART with cryopreserved sperm, the primary outcome measure is clinical pregnancy rate. Based on previous statistics from our reproductive medicine center, the overall clinical pregnancy rate for standard ART treatment is approximately 40%, which serves as the reference value (p0). Based on our center's statistical experience and previous literature reports (21), the estimated clinical pregnancy rate in the target population of this study is approximately 28% (p1). With a significance level *α* = 0.05 (two-sided) and power 1 − *β* = 0.90, using the “Proportions: Difference from constant (one sample case)” module for calculation, the minimum required sample size was determined to be 102 cases. The study ultimately enrolled 104 participants, meeting the calculated minimum sample size requirement and providing sufficient statistical power for the primary outcome measure.

### Statistical analysis

All statistical analyses were performed using R software (version 4.5.1). Continuous variables were presented as median (first and third quartiles) and mean ± standard deviation. The Shapiro–Wilk test revealed that most semen parameters (sperm concentration, percentage of progressively motile sperm, semen volume, normal morphology rate, DFI, and total progressively motile sperm count) deviated from a normal distribution within each abstinence duration group (all *P* < 0.05). Therefore, nonparametric methods were used for the subsequent between-group comparisons.

The World Health Organization (WHO) Laboratory Manual for the Examination and Processing of Human Semen (5th and 6th editions) explicitly specifies that the abstinence period prior to semen analysis should be maintained within the range of 2–7 days. Drawing on grouping strategies for abstinence duration reported in previous literature (e.g., three-group or four-group approaches) (10, 22), this study categorized abstinence periods into four intervals: 0–2 days, 3–5 days, 6–7 days, and >7 days.

The Kruskal–Wallis test was applied to compare the overall differences in each semen parameter across abstinence duration groups (0–2, 3–5, 6/7, and >7 days). When the overall difference was statistically significant (*P* < 0.05), Dunn's test was performed for pairwise comparisons, with *P*-values adjusted using Bonferroni correction. Boxplots with jitter points were generated to visualize data distributions and between-group significance.

Age was dichotomized into <40 and ≥40 years to evaluate the interaction between age and abstinence duration on DFI. An aligned rank transform (ART) was applied because the data did not meet the normality assumption for two-way ANOVA, followed by a two-way ANOVA model to examine the main effects of age, abstinence duration, and their interaction. When the interaction was significant, simple effect analyses were conducted using the emmeans package (comparing DFI across abstinence duration groups stratified by age), with Bonferroni adjustment.

For a clinically oriented analysis of prolonged abstinence on high DFI (threshold set at >25%), the proportion of samples with DFI > 25% was calculated for each abstinence group, and comparisons were made using chi-square test (or Fisher's exact test when expected frequencies were <5).

A generalized additive model was constructed to explore potential nonlinear relationships between abstinence duration and DFI and assess whether age modifies such relationships. In this model, DFI was the dependent variable, abstinence duration was the independent variable, and its smooth function was allowed to vary by age group [s (abstinence_duration, by = age_group)]. The restricted maximum likelihood method was used to select smoothing parameters. Predicted DFI trends across abstinence durations (1–30 days) along with 95% confidence intervals were generated and plotted for each age group. Locally weighted regression was used as an adjunct approach to visualize the trends.

All tests were two sided, and the significance level was set at *α* = 0.05.

## Result

### General characteristics of sperm across varying abstinence durations

**Figure 1:**
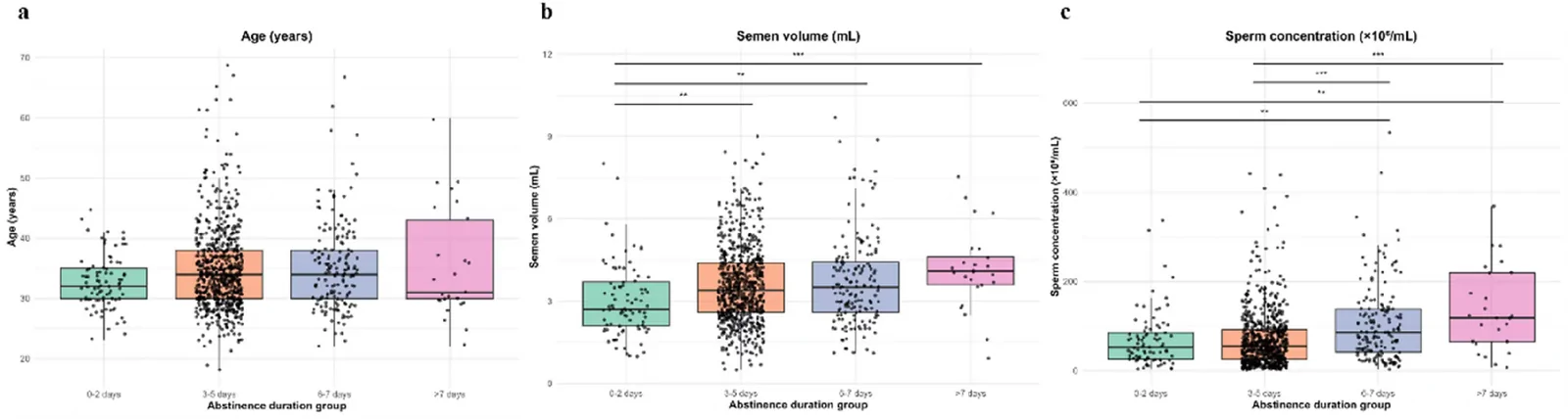
Characteristics of patients undergoing sperm acquisition across varying abstinence durations. Boxplot depicting age **(a)**, semen volume **(b)**, and sperm concentration **(c)** in patients undergoing sperm acquisition across varying abstinence durations. *, **, ***, and **** indicate significance at *p* < 0.05, *p* < 0.01, *p* < 0.001, and *p* < 0.0001, respectively.

**Figure 2:**
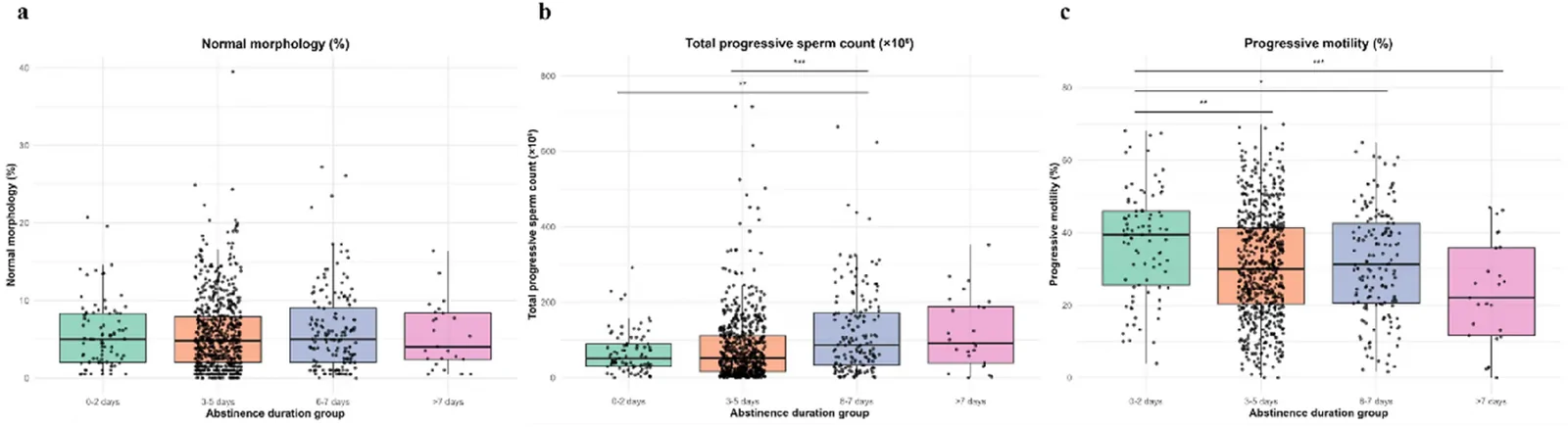
Characteristics of sperm across varying abstinence durations. Boxplot depicting the sperm's normal morphology **(a)**, total progressive sperm count **(b)**, and progressive motility **(c)** in patients undergoing sperm acquisition across varying abstinence durations. *, **, ***, and **** indicate significance at *p* < 0.05, *p* < 0.01, *p* < 0.001, and *p* < 0.0001, respectively.

The quality of sperm samples from the population who underwent sperm cryopreservation from January 2023 to December 2024 was analyzed and categorized into four groups on the basis of abstinence duration: 0–2, 3–5, 6/7, and >7 days. In accordance with established sperm cryopreservation guidelines, the majority of the cohort (673 individuals) completed the procedure within 3–5 days (Table 1). The Kruskal–Wallis test revealed no significant association between age distribution and abstinence duration (Table 2 and Figure 1a). However, semen volume (mL) and sperm concentration demonstrated significant correlations with abstinence duration. As the duration of abstinence lengthened, semen volume (mL) and sperm concentration (×10^6^/mL) exhibited a corresponding increase (Table 2 and Figures 1b,c). The quality of sperm in semen may vary depending on the duration of abstinence. The Kruskal–Wallis test revealed no significant association between normal morphological (%) distribution and abstinence duration (Table 2). However, progressive motility (%) and total progressive sperm count (×10^6^) demonstrated significant correlations with abstinence duration (Table 2). The proportion of sperm with normal morphology (%) did not differ significantly with the duration of abstinence (Figure 2a). Although the total progressive sperm count increased (Figure 2b), the progressive motility of sperms showed a decline as the duration of abstinence lengthened (Figure 2c).

**Table 1:** Semen parameters by abstinence duration [median (Q1–Q3)].

Abstinence group	*n*	DNA fragmentation index (DFI, %)	Total progressive sperm count (×10^6^)	Progressive motility (%)	Age (years)	Normal morphology (%)	Sperm concentration (×10^6^/mL)	Semen volume (mL)
0–2 days	85	11.8 [8.2–20.1]	51.0 [30.2–89.8]	39.5 [25.5–46.0]	32.0 [30.0–35.0]	5.0 [2.0–8.3]	53.3 [26.1–85.0]	2.7 [2.1–3.7]
3–5 days	673	15.8 [10.6–25.3]	52.2 [16.8–111.1]	30.0 [20.4–41.3]	34.0 [30.0–38.0]	4.8 [2.0–7.9]	54.6 [25.3–92.1]	3.4 [2.6–4.4]
6/7 days	159	18.1 [12.0–25.9]	86.2 [33.6–172.4]	31.3 [20.6–42.5]	34.0 [30.0–38.0]	5.0 [2.0–8.6]	86.1 [41.7–138.2]	3.5 [2.6–4.4]
>7 days	25	26.7 [22.7–41.7]	91.0 [39.4–188.4]	22.0 [11.6–35.8]	31.0 [30.0–43.0]	4.0 [2.4–8.4]	118.2 [65.1–219.3]	4.1 [3.6–4.6]

**Table 2:** Kruskal–wallis and dunn's *post-hoc* test results of semen.

Parameter	Kruskal–Wallis (p)	Significant pairs (Dunn)
Age (years)	6.08 × 10^−2^	—
Semen volume (mL)	<0.0001	0–2 days vs. 3–5 days (**); 0–2 days vs. 6/7 days (**); 0–2 days vs. >7 days (***)
Progressive motility (%)	<0.001	0–2 days vs. 3–5 days (**); 0–2 days vs. 6/7 days (*); 0–2 days vs. >7 days (***)
DNA fragmentation index (DFI, %)	<0.0001	0–2 days vs. 3–5 days (**); 0–2 days vs. 6–7 days (***); 0–2 days vs. >7 days (****); 3–5 days vs. >7 days (****); 6/7 days vs. >7 days (**)
Sperm concentration (×10^6^/mL)	<0.0001	0–2 days vs. 6/7 days (**); 0–2 days vs. >7 days (**); 3–5 days vs. 6/7 days (****); 3–5 days vs. >7 days (***)
Normal morphology (%)	9.67 × 10^−1^	—
Total progressive sperm count (×10^6^)	<0.0001	0–2 days vs. 6/7 days (**); 3–5 days vs. 6/7 days (****)

*, **, ***, and **** indicate significance at *p* < 0.05, *p* < 0.01, *p* < 0.001, and *p* < 0.0001, respectively (adjusted for multiple comparisons).

### Characteristics of DFI with abstinence duration and age

**Figure 3:**
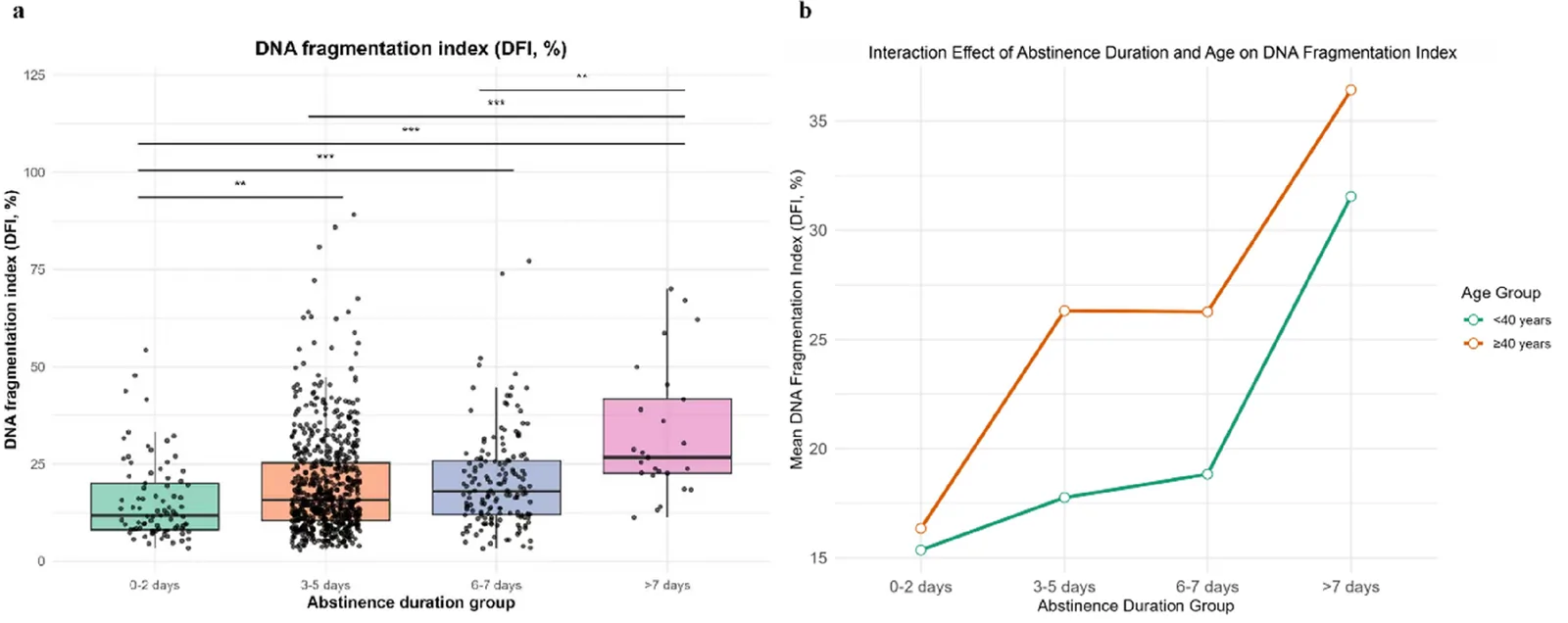
DFI of sperm across varying abstinence durations. **(a)** Boxplot depicting the DFI of sperm across varying abstinence duration and **(b)** line chart illustrating the changing trend of DFI in different age groups (< 40 and ≥40 years) across varying abstinence durations. *, **, ***, and **** indicate significance at *p* < 0.05, *p* < 0.01, *p* < 0.001, and *p* < 0.0001, respectively.

The findings indicated that the DFI in sperm increased as the duration of abstinence increased. Specifically, no significant difference was detected between the 3–5- and 6/7-day groups (Figure 3a), whereas a pronounced increase was observed when abstinence extended beyond 7 days (Figure 3a). The association between patient age and DFI were investigated, and the results demonstrated that patients over 40 years old generally exhibited higher DFI than those under 40 years old (Figure 3b). When the abstinence period was within 0–2 days, the age-related difference in DFI was not statistically significant. However, with prolonged abstinence, patients over 40 years old showed a marked increase in DFI (Figure 3b), which may contribute to reduced success rates in ART. The optimal timing for sperm retrieval in elderly patients remains a subject of debate, and earlier sperm cryopreservation, particularly within the first 0–2 days, may be advisable.

### Optimal timing for abstinence duration among older adults

**Figure 4:**
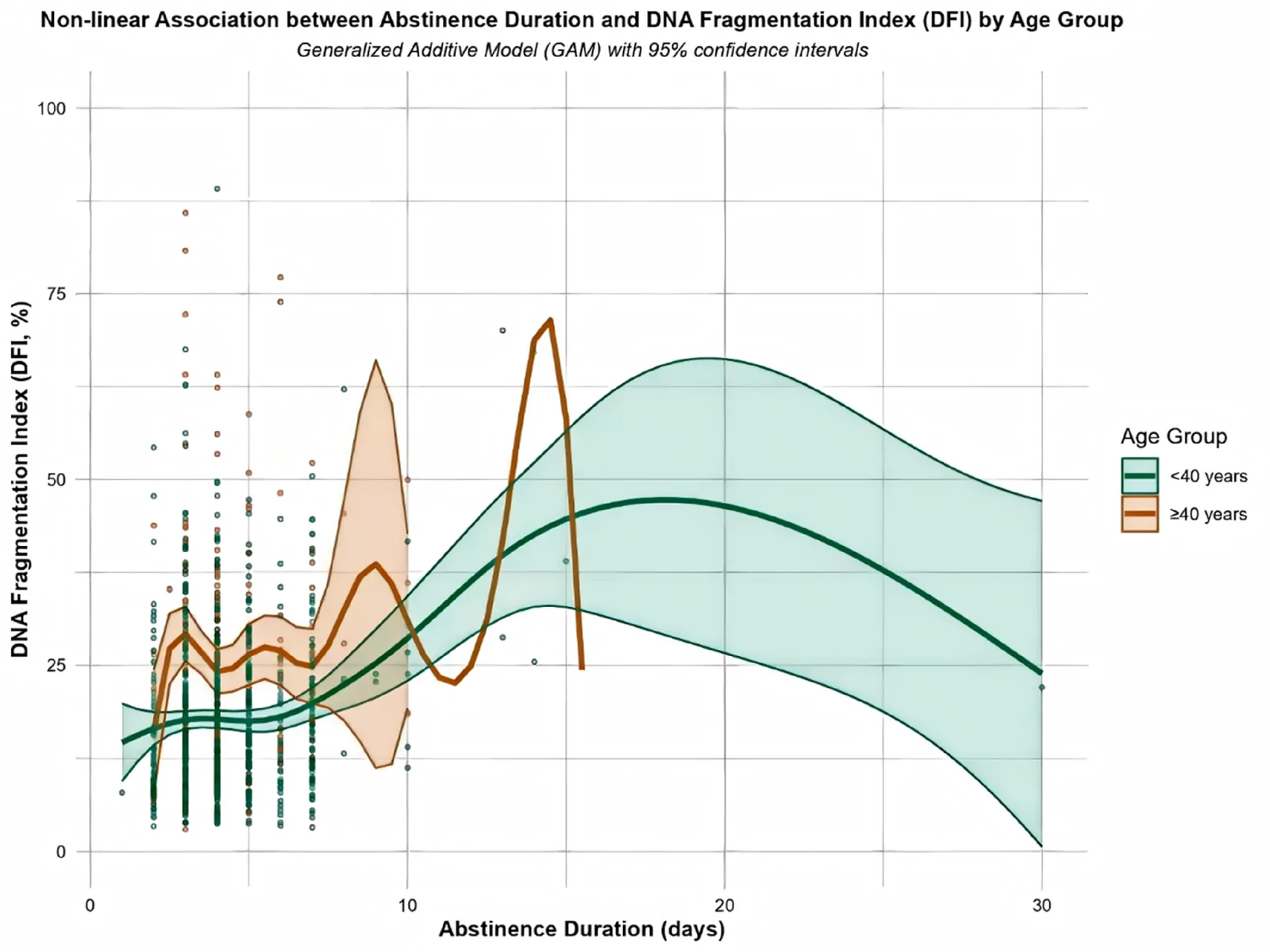
Nonlinear association between abstinence duration and DFI by age group.

A nonlinear correlation analysis was performed using existing cryopreserved semen data to further elucidate the influence of age and abstinence duration on sperm DFI. Predictive modeling of DFI trends with increasing abstinence time across different age cohorts revealed that in patients under 40 years of age, DFI exhibited a gradual increase with advancing age (Figure 4). By contrast, patients over 40 years of age displayed marked instability and irregular fluctuations in DFI, with significantly increased levels observed within the first 10 days of abstinence relative to their younger counterparts (Figure 4). These outcomes indicate heightened inter-individual variability in semen quality among older patients and suggest that DFI assessment becomes progressively more complex with extended abstinence. The results further corroborate the earlier conclusion that within older populations, shortening the abstinence period and collecting semen samples earlier can yield specimens of superior quality.

### Effects of sexual abstinence on assisted reproductive outcomes

A total of 104 patients who subsequently received ART were enrolled, and their reproductive outcomes were investigated. The mean age of the cohort was 38.96 years, with the ≥40- and <40-year age groups having mean ages of 43.52 and 33.65 years, respectively. A comparison of baseline characteristics between the two age-stratified groups revealed statistically significant differences in clinical features (*P* < 0.05, Table 3). Specifically, significant statistical disparities were observed between the two groups in terms of the wife's age, day-3 (D3) embryos, blastocysts, and live birth outcomes (*P* < 0.05, Table 3).

**Table 3:** Outcomes of assisted reproduction in age groups [median (Q1–Q3)].

Variable	*n* = 104^1^	Age		Statistics	*P*-value^2^
		<40 *n* = 48 (46%)^1^	≥40 *n* = 56 (54%)^1^		
Age	38.96 (5.95)	33.65 (3.66)	43.52 (3.00)	0.0	<0.001
Abstinence day	4.00 [2.50, 7.00]	4.00 [3.00, 6.50]	4.00 [2.00, 7.00]	1,273.5	0.64
Abstinence groups					0.69
0–2 days	26 (25.00%)	11 (22.92%)	15 (26.79%)		
3–5 days	37 (35.58%)	20 (41.67%)	17 (30.36%)		
6/7 days	30 (28.85%)	13 (27.08%)	17 (30.36%)		
>7 days	11 (10.58%)	4 (8.33%)	7 (12.50%)		
Wife's age	35.00 [32.50, 38.00]	34.00 [31.00, 36.00]	37.00 [35.00, 39.00]	674.5	<0.001
D3 embryo	3.00 [2.00, 5.00]	4.00 [3.00, 5.00]	3.00 [2.00, 4.00]	1,959.5	<0.001
Blastocyst	1.00 [0.00, 2.00]	1.00 [0.00, 2.00]	0.50 [0.00, 1.00]	1,680.0	0.020
Transplant	1.00 [1.00, 1.00]	1.00 [1.00, 1.00]	1.00 [1.00, 1.00]	1,256.0	0.22
Live birth	1.00 [0.00, 1.00]	1.00 [1.00, 1.00]	1.00 [0.00, 1.00]	1,809.0	<0.001

1 Mean (SD); Median [Q1, Q3]; *n* (%).

2 Wilcoxon rank sum test; Fisher's Exact Test for Count Data with simulated *p*-value (based on 2,000 replicates).

Abstinence duration was incorporated into the grouping to compare baseline characteristics. The <40-year age group generally exhibited more favorable reproductive outcomes, with no statistically significant differences in reproductive outcomes across varying abstinence durations (Table 4). By contrast, in the ≥40-year age group, shorter abstinence durations were associated with better reproductive outcomes, with a statistically significant difference noted in live birth outcomes (*P* < 0.001, Table 4).

**Table 4:** Assisted reproductive outcomes at different abstinence duration days in various age groups.

Variable	<40 years old							≥40 years old						
	*n* = 48^1^	0–2 days^1^	3–5 days^1^	6/7 days^1^	>7 days^1^	Statistics	*P*-value^2^	*n* = 56^1^	0–2 days^1^	3–5 days^1^	6/7 days^1^	>7 days^1^	Statistics	*P*-value^2^
Age	33.65 (3.66)	33.36 (3.44)	32.30 (3.28)	35.54 (4.16)	35.00 (1.63)	7.42	0.060	43.52 (3.00)	42.87 (3.20)	44.41 (2.79)	43.41 (3.30)	43.00 (2.31)	3.80	0.28
Abstinence day	4.00 [3.00, 6.50]	2.00 [2.00, 2.00]	4.00 [3.00, 4.00]	7.00 [6.00, 7.00]	8.50 [8.00, 9.50]	43.16	<0.001	4.00 [2.00, 7.00]	2.00 [2.00, 2.00]	4.00 [3.00, 4.00]	7.00 [6.00, 7.00]	8.00 [8.00, 9.00]	52.17	<0.001
Wife's age	34.00 [31.00, 36.00]	33.00 [31.00, 36.00]	34.00 [30.00, 35.00]	35.00 [31.00, 37.00]	36.00 [33.50, 40.00]	4.52	0.21	37.00 [35.00, 39.00]	36.00 [35.00, 39.00]	38.00 [35.00, 39.00]	37.00 [35.00, 39.00]	35.00 [30.00, 37.00]	3.15	0.37
D3 embryo	4.00 [3.00, 5.00]	4.00 [3.00, 6.00]	5.00 [3.50, 5.50]	4.00 [3.00, 4.00]	4.50 [2.50, 5.50]	3.03	0.39	3.00 [2.00, 4.00]	3.00 [2.00, 5.00]	3.00 [2.00, 4.00]	2.00 [2.00, 3.00]	2.00 [1.00, 4.00]	5.35	0.15
Blastocyst	1.00 [0.00, 2.00]	2.00 [1.00, 2.00]	1.50 [0.50, 2.00]	0.00 [0.00, 1.00]	1.00 [0.00, 2.00]	5.59	0.13	0.50 [0.00, 1.00]	1.00 [0.00, 1.00]	1.00 [0.00, 2.00]	0.00 [0.00, 1.00]	0.00 [0.00, 1.00]	3.41	0.33
Transplant	1.00 [1.00, 1.00]	1.00 [1.00, 1.00]	1.00 [1.00, 1.00]	1.00 [1.00, 1.00]	1.00 [1.00, 1.00]	1.41	0.70	1.00 [1.00, 1.00]	1.00 [1.00, 1.00]	1.00 [1.00, 1.00]	1.00 [1.00, 1.00]	1.00 [1.00, 2.00]	4.42	0.22
Live birth	1.00 [1.00, 1.00]	1.00 [1.00, 1.00]	1.00 [1.00, 1.00]	1.00 [1.00, 1.00]	1.00 [0.50, 1.00]	4.60	0.20	1.00 [0.00, 1.00]	1.00 [1.00, 1.00]	1.00 [1.00, 1.00]	0.00 [0.00, 0.00]	0.00 [0.00, 1.00]	23.17	<0.001

1 Mean (SD); Median [Q1, Q3].

2 Wilcoxon rank sum test; Fisher's Exact Test for Count Data with simulated *p*-value (based on 2,000 replicates).

In conjunction with the aforementioned findings, this study further validated that shortening abstinence duration can considerable improve semen quality in elderly patients, ultimately contributing to favorable reproductive outcomes.

## Discussion

The peak incidence ages of various diseases differ, resulting in distinct age distributions among patients seeking sperm cryopreservation. Furthermore, as certain patients with cancer require prompt initiation of radiotherapy or chemotherapy, no universally standardized protocol is available for the abstinence period before semen preservation. This challenge is particularly evident in clinical practice, where the scheduling of radiotherapy and chemotherapy must often be tailored to the patient's specific condition and therapeutic regimen. For individuals undergoing sperm cryopreservation, completing the procedure within a constrained timeframe presents a considerable practical difficulty. Thus, healthcare teams should take into comprehensive account factors, such as disease type, age at onset, and treatment timeline, when formulating therapeutic plans to devise optimal fertility preservation strategies. In recent years, the relationship between semen quality and abstinence duration in the context of sperm cryopreservation has attracted increasing attention. The influence of abstinence length on semen quality involves multiple physiological and biochemical mechanisms, rendering it a relatively complex scientific issue.

The results of this study indicated that with prolonged abstinence duration, semen volume and sperm concentration showed an upward trend. However, while longer abstinence periods contributed to an increase in sperm count, sperm motility tended to decline, reflecting the complex influence of abstinence duration on semen parameters. First, extended abstinence is indeed associated with increases in semen volume and concentration. A systematic review and meta-analysis noted that longer abstinence periods corresponded to higher sperm concentration and semen volume (23). However, an increase in quantity does not equate to an improvement in quality because sperm motility and other functional indicators may be adversely affected. Second, although sperm count increases with prolonged abstinence, sperm motility may decrease. Longer abstinence is associated with reduced sperm motility, which may result from prolonged retention of sperm in the reproductive tract, leading to increased oxidative stress and DNA fragmentation levels (23, 24). Other studies have shown that shorter abstinence periods are associated with a lower sperm DFI and higher sperm motility, suggesting that appropriately reducing abstinence duration may help improve sperm functional performance (6, 24). Finally, the effect of abstinence duration on semen parameters may vary depending on individual differences and clinical context. For example, in patients with infertility, longer abstinence may lead to further deterioration in sperm motility and morphology, whereas shorter abstinence may be beneficial for improving related parameters (9, 25). Therefore, optimizing the abstinence period based on individual circumstances is recommended to achieve the best reproductive outcomes.

This study revealed a significant association between prolonged abstinence and increased sperm DNA fragmentation, with the trend being more pronounced in individuals over 40 years of age. Research has indicated that DNA fragmentation notably increases after more than 4 days of abstinence (11). By contrast, shorter abstinence periods are not only associated with lower DNA fragmentation rates but also correlated with improved pregnancy and live birth rates (26). Regarding the factor of age, sperm DNA fragmentation tends to increase with advancing male age, and this finding may be related to increased oxidative stress levels in the body (27). Specifically, men over 40 years of age face a considerable higher risk of sperm DNA damage (28). Further studies have shown that sperm DNA fragmentation rates are markedly higher in men over 35 years than in younger groups, underscoring the need to consider male age in infertility assessments (29). Additionally, short-term abstinence has been demonstrated to effectively reduce DNA fragmentation. For instance, a significant decrease in fragmentation rates can be observed after only 3–4 h of abstinence (30), and another study supports that abstinence for 1 day can effectively improve this parameter (7). The present study demonstrated that elderly male patients exhibited superior sperm quality parameters (including progressive motility and normal morphology rates) following shorter abstinence durations (0–2 and 3–5 days), with a concurrent upward trend in live birth rates among those undergoing ART. These findings collectively indicated that short-term abstinence is an effective strategy for improving sperm quality. On the basis of current evidence, individuals over 40 years of age are recommended to consider limiting abstinence to within 0–2 days to optimize reproductive health outcomes. A short abstinence duration remarkably enhanced total sperm motility and progressive motility while reducing sperm DNA fragmentation (14). Furthermore, in patients with severe oligoasthenozoospermia, the second semen sample collected following a short abstinence period exhibited substantial improvements in clinical pregnancy rates and embryo quality (13). These findings suggest that shortening the abstinence duration may represent an effective strategy to improve the success rate of ART. However, due to the limited sample size of elderly patients, the association between semen parameters and final reproductive outcomes in this population remains unclear. Additionally, there is a lack of systematic follow-up data on the long-term health status of their offspring after birth, which warrants further in-depth exploration through multicenter prospective studies.

In summary, this study analyzed semen quality data from patients enrolled in the sperm bank's cryopreservation program to identify the ideal abstinence duration across different age groups. The findings revealed that among patients over 40 years old, the sperm DFI increased with longer abstinence periods, whereas the overall sperm quality exhibited an inconsistent pattern. Accordingly, moderately reducing the duration of abstinence in this population is advisable. Subsequent research should involve more refined age stratification and abstinence intervals, along with the inclusion of additional relevant factors, to offer more precise recommendations for pre-cryopreservation abstinence timing in patients of varying age groups.

### The limitations of this study

The study is subject to several limitations. First, all samples and data were derived from one reproductive medicine center. This single-center cohort lacks multicenter validation, and regional population-specific characteristics, including local lifestyles, dietary patterns, and the etiological spectrum of infertility, may restrict the generalizability of the stratified abstinence protocol to populations of other ethnicities or geographic regions. Second, due to partial missingness of clinical data, comprehensive patient information collection was not fully achieved. Future studies should supplement multivariable regression analyses adjusted for lifestyle-related confounders [e.g., smoking status, body mass index (BMI), alcohol consumption, and duration of infertility] to enhance the evidence strength regarding the independent interactive effects of age and abstinence duration on DFI. Third, refinement of age stratification is required. However, given the limited clinical sample size in the current study, more granular age grouping would result in insufficient sample sizes per subgroup, precluding subsequent statistical analyses. Expansion of the sample size in future studies is thus necessary to enable more detailed age categorization. Finally, the potential improvement in offspring safety associated with shorter abstinence periods in older men remains uncertain. Future multicenter prospective cohort studies, coupled with long-term follow-up of neonatal outcomes, are warranted to validate the efficacy of the stratified abstinence strategy.

## Data Availability

The raw data supporting the conclusions of this article will be made available by the authors, without undue reservation.
